# Epigenetic modulation by life–style: advances in diet, exercise, and mindfulness for disease prevention and health optimization

**DOI:** 10.3389/fnut.2025.1632999

**Published:** 2025-08-21

**Authors:** José Ostaiza-Cardenas, Andrea C. Tobar, Stefany Carolina Costa, Diana Sofia Calero, Alisson López-Carrera, Fátima German Bermúdez, Andrea Orellana-Manzano

**Affiliations:** ^1^School of Medicine, Medical Sciences Faculty, Universidad de Guayaquil, Guayaquil, Ecuador; ^2^School of Medicine, Pontificia Universidad Católica del Ecuador, Quito, Ecuador; ^3^Laboratorio para Investigaciones Biomédicas, Facultad de Ciencias de la vida, Escuela Superior Politécnica del Litoral (ESPOL), Campus Gustavo Galindo, Guayaquil, Ecuador; ^4^Facultad de Ciencias de la Vida, Escuela Superior Politécnica del Litoral, Campus Gustavo Galindo, Guayaquil, Ecuador

**Keywords:** epigenetic mechanisms, mindfulness, nutrition, physical activity, health, DNA methylation

## Abstract

Socio-economic and environmental factors significantly influence health by driving epigenetic changes that alter genetic expression and impact disease prevention. Lifestyle elements such as diet, exercise, mindfulness, and environmental exposure play crucial roles in modulating these mechanisms. A systematic review of studies from the past 13 years, conducted under PRISMA guidelines, examined interventions, epigenetic outcomes, and health impacts. Mindfulness practices, particularly meditation, were found to regulate DNA methylation, reducing stress and inflammation. Dietary interventions, such as the Mediterranean and DASH diets, enhanced health biomarkers and slowed epigenetic aging through favorable DNA methylation. Physical activities, such as high-intensity interval training and hybrid training, induced epigenetic modifications, improving metabolic function, mitochondrial biogenesis, and insulin sensitivity. These findings emphasize the importance of adopting modern lifestyle choices to promote health and prevent chronic diseases by influencing gene expression. Combining mindfulness, balanced diets, and regular physical activity offers substantial benefits for metabolic, cardiovascular, and mental health. However, more research is needed to understand the long-term effects of lifestyle factors on epigenetics and to develop personalized strategies that optimize disease prevention and overall wellbeing.

## 1 Introduction

Epigenetics refers to modifications in gene expression that occur without changing the DNA sequence. These changes can be heritable but shift dynamically in response to environmental factors such as nutrition, physical activity, stress, exposure to pathogens or pollution, sleep quality, social environment, and lifestyle changes. Epigenetic modifications are maintained throughout various cycles of cell division but can also be reversed (1).

Scientists have discovered three key epigenetic processes: methylation of DNA, modifications to histones, and gene silencing through non-coding RNAs (ncRNAs) (2). Among all the epigenetic changes, DNA methylation, first described in 1965, is the most studied mechanism and stable epigenetic marker. DNA methylation occurs when a methyl group is attached to carbon-5 of cytosine bases, specifically within CpG dinucleotides (3). This process is associated with gene silencing in the CpG islands of promoter regions because of reduced interaction between DNA and transcription factors. In most cases, higher levels of methylation in a gene's regulatory region lead to decreased activity, while reduced methylation promotes gene activity, thereby influencing gene expression. One of the most extensively researched clinical applications of epigenetic mechanisms is in the field of cancer, where hypermethylation of tumor suppressor genes and hypomethylation of proto-oncogenes are commonly observed. For example, there is evidence that DNA methylation decreases as a benign tumor develops into invasive cancer (2, 4).

Histone alterations can promote gene expression by enabling the interaction between transcription factors and enzymes with DNA or suppress gene expression by inhibiting the initiation of transcription. These post-translational modifications to histone proteins consist of acetylation, methylation, ubiquitylation, and phosphorylation, all catalyzed by enzymes that will modify how DNA interacts in nucleosomes (2).

Non-coding RNA-associated gene silencing is the most recently identified epigenetic mechanism. Non-coding RNA (ncRNA), which are transcribed but unable to be translated into proteins, were once thought to be non-functional remnants of the genome. However, emerging evidence highlights their critical role in regulating epigenetic gene expression, especially in DNA methylation, histone modification, and gene silencing. Epigenetics is a promising research domain because it can modulate gene expression without altering the DNA sequence (2).

Lifestyle factors significantly influence epigenetic changes, which are modifications in gene expression that do not involve alterations to the DNA sequence. For instance, dietary components, such as folate and polyphenols, can affect DNA methylation and histone modifications, thereby regulating gene expression related to health outcomes, including cancer and metabolic disorders. A study by Malekina et al. (5) has shown that adequate intake of these nutrients can lead to favorable epigenetic changes, while deficiencies may result in increased disease susceptibility (6).

Physical activity also plays a crucial role in shaping the epigenome. Regular exercise has been associated with beneficial alterations in DNA methylation patterns that enhance metabolic health and reduce the risk of chronic diseases. Research indicates that exercise can induce changes in genes involved in inflammation and metabolism through epigenetic mechanisms, promoting overall health and longevity (7). In contrast, unhealthy lifestyle choices, such as smoking and excessive alcohol consumption, have been linked to adverse epigenetic modifications that contribute to disease progression (8, 9).

**Figure 1:**
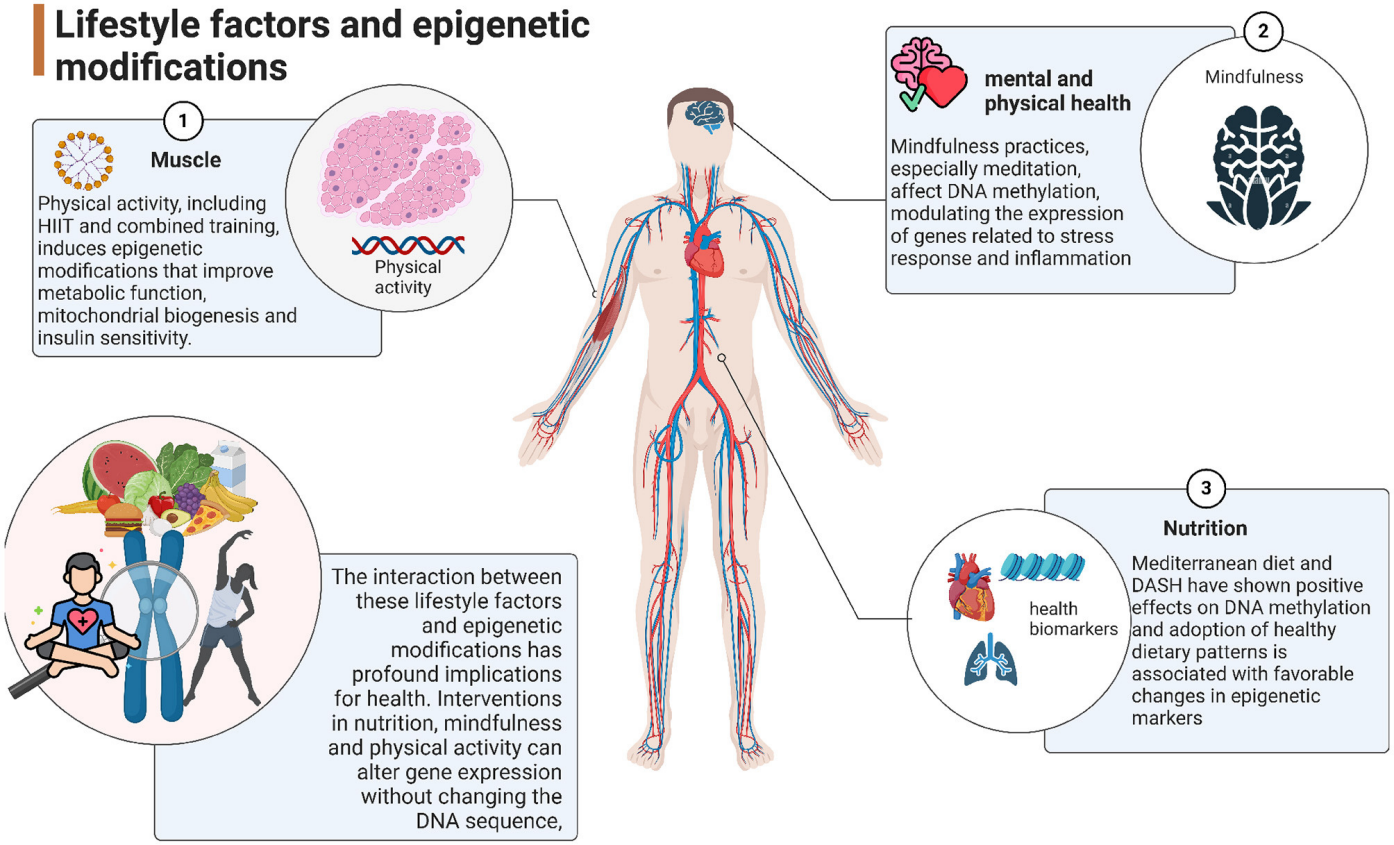
Graphical abstract of lifestyle factors and epigenetic modifications.

Moreover, environmental factors such as exposure to pollutants and psychosocial stress can lead to significant epigenetic changes (10, 11). Stress has been shown to alter the expression of genes involved in stress response pathways through mechanisms such as DNA methylation (12, 13). Similarly, exposure to environmental toxins can disrupt normal epigenetic regulation, increasing the risk of various health issues (14–16). Understanding how lifestyle factors influence epigenetic changes is vital for developing preventive strategies against diseases and promoting better health outcomes across populations (Figure 1).

## 2 Materials and methods

This systematic review adheres to the Preferred Reporting Items for Systematic Reviews and Meta-Analyses (PRISMA) guidelines (142). The focus of the evaluation was to examine the impact of modern lifestyle activities on epigenetic markers, drawing on studies sourced from PubMed, ScienceDirect, Scopus, Cochrane, Clinical Trials, and Google Scholar databases. Articles were excluded if published over 13 years ago, were not indexed in the selected databases, or did not meet the specified inclusion criteria.

The selection process occurred in two phases. In the first phase, the titles and abstracts of potential studies were reviewed to identify those that met the inclusion criteria, and those that did not meet the criteria were excluded. In the second phase, full-text articles were reviewed in detail to confirm eligibility based on the established criteria.

A customized search strategy was developed for each database, focusing on original research articles published within the last 13 years. Data collection took place over 21 days, from 7th October to 26th October, with subsequent analysis conducted from 27th October to 3rd November. For each included study, the following information was extracted: author(s), year of publication, target age group and characteristics, type of lifestyle intervention implemented, epigenetic mechanisms assessed, results, and the specific parameters evaluated. The authors independently reviewed all full-text articles.

### 2.1 Eligibility criteria

#### 2.1.1 Inclusion and exclusion criteria

Studies were considered eligible for inclusion if they met the following criteria: (1) original research articles; (2) involved a change in lifestyle activities, such as physical activity, nutrition, mindfulness, or outdoor activities; (3) focused on modern lifestyle interventions with a delayed impact; (4) assessed epigenetic biomarkers; and (5) involved human subjects. Studies were excluded if they were published more than 13 years ago, involved non-human models, were purely theoretical, were books, or did not align with the review's objectives.

#### 2.1.2 Search strategies

To enhance the search, specific keywords were used, such as “Lifestyle” OR “mindfulness” OR “Diet” OR “ketogenic diet” OR “veganism” OR vegan diet” OR “intermittent fasting” OR “hybrid training” OR “combined training” OR “strength training” OR “resistance training” OR “High- Intensity Interval Training” OR “High Intensity training” OR “High Intensity” OR “HIIT” AND “epigenetic biomarkers” OR “epigenetic clock” OR “epigenetic” OR “DNA methylation” OR “Histones” OR “mRNA” OR “telomere” OR “genetic” OR “epigenome”.

## 3 Results

### 3.1 Study selection

A comprehensive search in the PubMed electronic database initially yielded 207 studies. After reviewing the abstracts, 192 articles were excluded. Additionally, a search on Google Scholar identified 67,180 articles, with 7,000 screened within the designated timeframe. Of these, 6,964 were excluded because they did not meet the criteria, and an additional 12 were excluded after further examination, leaving 24 studies that fulfilled the final inclusion criteria. Finally, a search on ScienceDirect identified 167,000 results, with 1,000 screened. This review includes 39 references.

Mindfulness: The bibliographic search was carried out in Cochrane, Clinical Trials, and PubMed with the keywords “strength mindfulness,” and “epigenetic”, 22, 6, and 70 articles, respectively; 98 articles were found from 2011 to 2024, and analyzing each article, 14 articles were found that were used in this research and met the criteria of inclusion. Finally, only 14 articles met the inclusion criteria.

Physical activity: The bibliographic search was conducted in Google Scholar using the keywords “strength training” and “epigenetics,” yielding 17,900 articles from 2019 to 2024. After analyzing each article, 11 articles were selected for this research and met the inclusion criteria. The bibliographic search was conducted in PubMed using the keywords “strength training” and “epigenetics”; 38 articles were published between 2019 and 2024, of which only two met the inclusion criteria. Finally, only 28 articles met the inclusion criteria.

**Figure 2:**
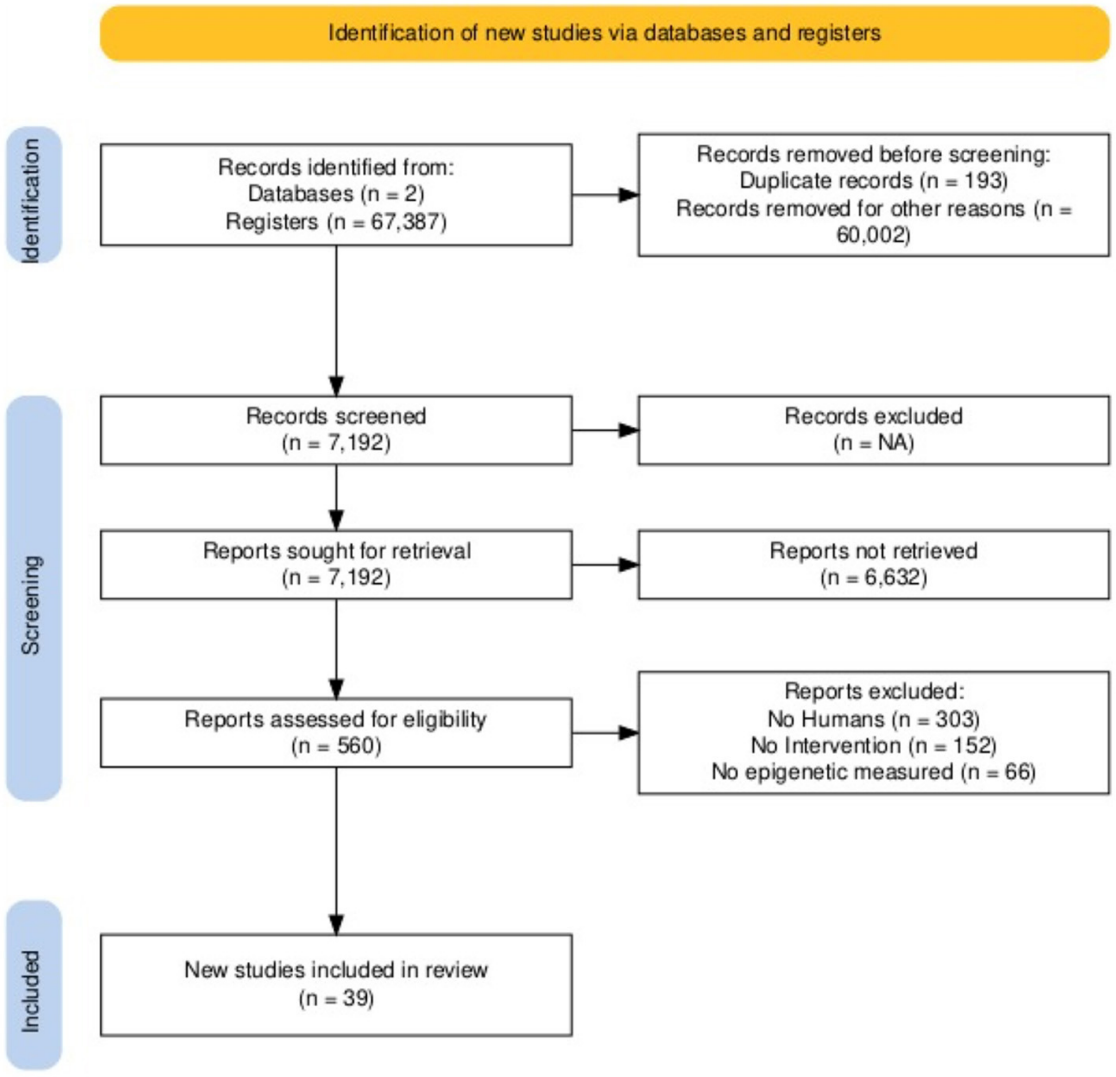
Study design of the selection process for studies on mindfulness, nutrition, and physical activity influences epigenetic changes.

Nutrition: The bibliographic search was conducted in Cochrane, Clinical Trials, and PubMed using the keywords “strength nutrition” and “epigenetics,” yielding 154, 29, and 6,739 articles, respectively. A total of 6,922 articles were identified between 2011 and 2024. Analyzing each article, 21 articles were found that were used in this research and met the inclusion criteria. Finally, only 21 articles met the inclusion criteria (Figure 2).

### 3.2 Study characteristics

We divided the information into three main tables, which are submitted as Supplementary Tables. Supplementary Table 1 shows studies on the effects of mindfulness-based therapy and its impact on epigenetic changes, specifically on DNA methylation. It details characteristics, such as title, population, characteristics of the population, country, type of intervention, parameters of mindfulness, epigenetic variation, epigenetic mechanisms, and results. Supplementary Table 2 summarizes the key characteristics of the studies analyzed in this research. These studies were conducted in different countries and published between 2019 and 2024. All the selected studies focused on the effects of applying a protocol where they could have been a diet protocol, physical activity (resistance training, high intensity or combined training), and the outcomes of these in biomarkers and epigenetics, such as DNA methylation, telomerase activity and telomere length, and expression of mRNA or the expression of proteins related with mitochondrial activity. Supplementary Table 3 shows the effects of nutrition therapy and its impact on epigenetic changes. It details characteristics, such as title, population, characteristics of the population, country, type of diet, parameters of diet, epigenetic variation, epigenetic mechanism, and results.

### 3.3 Data extraction

For each study in this review, the following information was extracted: sample, characteristics of the group (age, ethnicity, biometrics markers, groups with a disease, etc.), country, type of intervention (kind of mindfulness, type of physical activity, and type of diet intervention), epigenetic variation, epigenetic mechanism studied, and the main results observed.

#### 3.3.1 Mindfulness and epigenetics

Mindfulness involves cultivating non-judgmental awareness of one's thoughts, emotions, and surroundings in the present moment, with the intention of promoting clarity, calmness, and acceptance. By fostering an observational stance, mindfulness helps individuals interrupt automatic or harmful behavior patterns and encourages a deeper understanding of their internal states. It is integrated into various therapeutic modalities, including mindfulness-based cognitive behavioral therapy, mindfulness-based stress reduction, and mindfulness meditation, to enhance emotional regulation and overall wellbeing (17). Evidence shows that it offers a range of benefits for mental and physical wellbeing, including reductions in stress, psychological distress, depression, and certain aspects of burnout. It has been shown to enhance resilience, improve athletes' flow states, attention, and sleep, and support emotional regulation. Additionally, mindfulness may have physiological effects, potentially influencing markers of inflammation, cell-mediated immunity, and biological aging. It has also effectively attenuated pain and improved body image, particularly among women. These diverse benefits suggest that mindfulness is a valuable tool for enhancing both mental and physical health (18–25).

There are a variety of techniques in mindfulness: Mindful meditation involves non-judgmental attention to thoughts, emotions, and bodily sensations. The body scan focuses on awareness of bodily sensations to induce relaxation, while mindful movement integrates physical activity with focused attention, as seen in yoga, tai chi, and walking meditation (147). Loving-Kindness (Metta) meditation promotes compassion and empathy toward oneself and others through repeated mantras. Transcendental meditation uses silent mantras for deep relaxation and self-awareness. Guided imagery involves listening to visualizations to evoke calm, and mindful breathing emphasizes focused attention on the breath. Intensive mindfulness practices involve prolonged meditation for deeper self-inquiry, while daily activity mindfulness encourages presence and non-judgment in routine tasks (22, 25–27).

##### 3.3.1.1 Mechanisms of epigenetic modulation by mindfulness

The main categories of epigenetic mechanisms of gene regulation are DNA methylation, histone post-translational modifications, and the silencing action of non-coding RNAs. DNA methylation patterns impact gene expression in the brain and blood (28).

This study compares two groups of preclinical medical students from Heidelberg: one receiving a mindfulness-based stress reduction program for 3 months and a control group with no intervention. The program includes theoretical foundations on mindfulness, psychoeducation on stress and related disorders, and practical exercises such as mindful breathing, body scanning, and both sitting and walking meditation, complemented by feedback sessions to apply what was learned in daily life. The research assesses multiple variables, including biological measurements (SLC6A4 gene methylation, cortisol, and alpha-amylase) and psychological assessments (stress levels, mindfulness, and coping strategies), as well as other factors such as resilience, psychiatric symptoms, sleep quality, and self-compassion. Participants must be over 18 years of age, have access to the internet, and give written informed consent. All measurements, except for genetic polymorphisms, are taken before and after the intervention, and measured only once.

This study investigated the feasibility and effectiveness of a mindfulness-based intervention in adolescents with early life stress (ELS). Forty adolescents were randomly assigned to two groups: one that received eight sessions of Mindfulness-Based Stress Reduction for Adolescents (MBSR-T, *n* = 21) and a treatment-as-usual control group (CTRL, *n* = 17). The research measured biomarkers of stress (cortisol, C-reactive protein, and interleukin 6) and depressive symptoms before and after the intervention. The results showed that 16 of the 21 adolescents completed the program, with an average attendance of 6.5 sessions, and mean effects were observed in the reduction of depressive symptoms (Cohen's *d* = 0.69) and a trend toward lower levels of anticipatory cortisol (Cohen's *d* = 0.56) in the MBSR-T group compared to the control. However, no significant effects were found in inflammatory markers. The study demonstrated that it is feasible to implement group mindfulness interventions in adolescents with SLE and suggests possible benefits at both symptomatic and biological levels. However, more extensive studies are needed to confirm its efficacy.

##### 3.3.1.2 Impact of mindfulness on health

Mindfulness practices have been shown to significantly impact health by reducing stress, controlling inflammation, and improving mental wellbeing. Research indicates that mindfulness-based interventions, such as Mindfulness-Based Stress Reduction (MBSR), lead to substantial decreases in perceived stress, anxiety, and depression among participants, enhancing overall life satisfaction and wellbeing (29, 30). These practices foster a greater sense of calm and contribute to physiological benefits, such as lower inflammation levels, which are crucial for preventing chronic diseases (31). As mindfulness continues to gain traction across various sectors, including healthcare and education, its integration into routine practices may offer a viable strategy for promoting mental health and resilience in high-pressure environments (32, 33).

This research article and similar studies show the impact of stress on DNA, which has been linked to changes in the methylation of specific genes, which can influence gene expression and, thus, behavior (10). In a study on chronic social stress, differentially methylated regions (DMRs) were identified in the genome of mice exposed to stressful situations. Two of these regions include an intergenic area on the X chromosome, where a significant increase in methylation was observed in stressed mice, and intron 9 of the Drosha gene showed a reduction in methylation under stress conditions. These epigenetic changes may be related to disorders such as major depression since altered methylation in these regions was replicated in the post-mortem brains of patients with this condition. In addition, other genes involved in the stress response include those that are part of the hypothalamic-pituitary-adrenal (HPA) axis, such as corticotropin-releasing factor (CRF) and vasopressin, whose expression has been altered by chronic stress. Decreased methylation at the promoters of these genes may lead to an increase in their expression, contributing to behaviors related to anxiety and depression (10, 12).

Therefore, mindfulness helps lower systolic blood pressure and reduce stress levels. As a result, individuals are more likely to adopt healthier behaviors, thereby decreasing their risk of cardiovascular disease (34). Moreover, mindfulness has a substantial impact on mental health by playing a role in treating addictions by helping individuals stay present and aware of their current situation rather than diverting their attention to unhelpful or harmful distractions (35); increases individuals' capacity to observe and experience strong emotions with greater objectivity; and helps mitigating consequences of traumatic stress experiences by strengthening resilience (36).

##### 3.3.1.3 Studies on epigenetic changes induced by mindfulness

Over the past decade, there has been a growing interest in how psychological practices, such as mindfulness, may influence fundamental biological processes, including epigenetic regulation. Epigenetics—particularly DNA methylation—plays a central role in modulating gene expression without altering the underlying genetic sequence. Zannas and West suggest that mindfulness-based interventions may modify methylation patterns in genes associated with stress, inflammation, and cellular aging. Although this line of inquiry remains emerging, it presents significant implications for both mental and physical health (37). Studies published in the last 5 years have shown associations between regular meditation practice and changes in epigenetic markers. For example, a 2022 longitudinal study evaluated the differences in DNA methylation profiles among individuals who practiced mindfulness meditation during a 3-month intensive retreat. The results revealed significant reductions in the methylation of genes related to the inflammatory response, particularly in NF-κB and other cellular stress mediators (38). These changes were correlated with subjective improvements in emotional wellbeing. They reduced peripheral inflammation markers, such as high-sensitivity C-reactive protein (hs-CRP). The meta-analysis by Goyal et al. published in *Translational Psychiatry* (39) compared epigenetic profiles between long-term meditators and control subjects without prior meditation experience. Consistent differences were found in the methylation of genes involved in regulating the hypothalamic-pituitary-adrenal (HPA) axis, suggesting a possible molecular modulation of the stress response (39). However, these studies do not establish direct causality but rather highlight associations that require further experimental exploration.

Another study about the intense mindfulness meditation can impact the methylation profile at 61 CpG sites. This was observed in adults between 38 and 60 years of age from France in a study where DNA methylation profiles were analyzed at 485,512 sites across the genome for all samples. This type of intervention showed differential methylation in biological pathways that are remarkable for the inflammatory and immune systems where five transcription factors were involved (KLF15, EGR1, EGR2, SP3, and SP4). Additionally, this study suggests that methylation changes in fatty acid metabolism genes, such as ACADM, CPT1A, and HSD17B4, play a role in enhancing immune function through stress reduction strategies. Differential methylation in the DNA damage response pathway, as well as changes associated with telomere biology and biological aging, were also observed. Genes reported as potential blood biomarkers of mental health, such as PACSIN1 and SPTBN1 (associated with addiction-related behavior), GPR19 (associated with depression), and AUTS2 (associated with autism), were highlighted in the STRING analysis of this study (38).

This clinical trial in São Paulo, Brazil, evaluates the effect of a mindfulness-based intervention on DNA methylation, cognitive functions, stress response, and wellbeing in healthy adult women. The study compares two groups for 8 weeks: one group receives mindfulness training (2 h per week of meditation) and another active control group receives lectures (2 h per week). Participants are randomly assigned to the groups in a 1:1 ratio after a 1–3-week screening period, where their eligibility is determined, and they sign an informed consent form (40). In the same direction, this study at Süleyman Demirel University investigates how Basic Body Awareness Therapy (BBAT) and aerobic exercise affect telomere length in young adults with high-stress levels. The research compares these two types of interventions based on the premise that stress and depression can alter telomeric structure through increased pro-inflammatory cytokines. It examines epigenetic mechanisms, precisely telomere length, and DNA methylation, hypothesizing that BBAT, by its focus on stress management based on mind-body principles, could achieve changes in telomeric length in a shorter period than other approaches. Telomeres, which naturally shorten with each cell replication cycle, are influenced by both internal factors (such as DNA methylation) and external factors (such as lifestyle and exercise). This study seeks to determine what type of intervention is most effective in preserving their healthy structure (41).

Despite progress, several methodological limitations persist. Many studies lack proper randomization, active controls, or sufficient sample sizes to generalize findings. Additionally, confounding factors, such as diet, exercise, traumatic history, or genetic variability, are not always accounted for in analyses. A recent report emphasized that only 20% of published studies to date use rigorous longitudinal designs with follow-ups exceeding 6 months, making it difficult to interpret the long-term effects of mindfulness on epigenetics (42).

**Figure 3:**
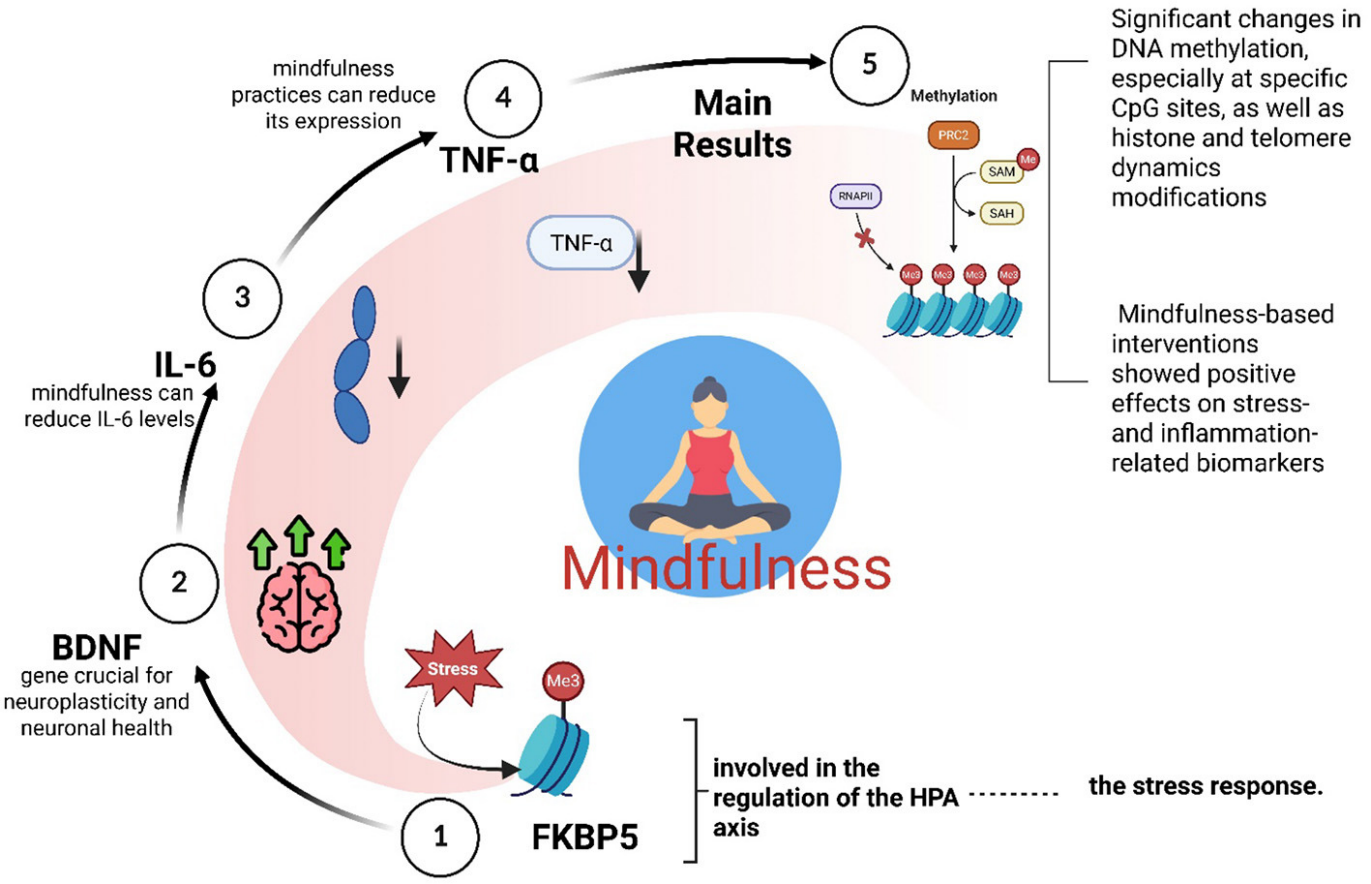
Epigenetic modulation by mindfulness.

The convergence of neuroscience, psychology, and genomics opens new opportunities to understand how mental practices can impact human biology. Future studies should incorporate multiomic techniques (genomics, transcriptomics, and metabolomics) alongside artificial intelligence tools to identify predictive patterns of mindfulness-induced epigenetic change. International collaborative projects, such as the “Mindfulness and Epigenetic Consortium” (MEC), are working toward this goal by developing large and diverse cohorts to validate preliminary observations (43) (Figure 3).

#### 3.3.2 Nutrition and epigenetics

Nutrition can play a role in generating epigenetic modifications and reversing epigenetic markers associated with disease. Studies indicate that having a low BMI, engaging in exercise, and consuming fish, poultry, fruits, and vegetables reduce the progression of the epigenetic clock. The anticancer trait of carotenoids stems from the DNA methylation of genes responsible for proangiogenic processes. Polyphenols can counteract adverse epigenetic regulation by modifying epigenetic markers, leading to the reactivation of beneficial genes (such as silenced tumor suppressor genes, antioxidant genes, and DNA repair genes) or deactivating detrimental genes (including oncogenes associated with inflammation, cell cycle progression, proliferation, invasion, angiogenesis, and metastasis). This positive epigenetic-mediated effect of polyphenols on the organism inhibits the pathogenesis of cardiovascular disease, obesity, metabolic syndrome, and cancer. Omega-3 fatty acids and selenium's anti-inflammatory and hypotriglyceridemic effects have also been linked to DNA methylation and microRNAs (44).

##### 3.2.2.1 Mediterranean diet and epigenetics

The Mediterranean diet is the traditional dietary pattern observed among the inhabitants of the Mediterranean region, characterized by the intake of different non-starchy vegetables, seeds, nuts, marginally refined whole-grain cereals, legumes (45), virgin olive oil, blue fish, and red wine. The epigenome has been identified as a key target for modulating gene expression influenced by bioactive nutrients from foods, such as polyphenols, which donate methyl groups, retinoids, and isothiocyanates. The low animal protein intake and the low glycemic index of this diet modulate the mammalian target of the rapamycin (mTOR) pathway, and the level of insulin-like growth factor-1 (IGF1), consequently leading to the activation of FOCO3A and finally to the transcription of homeostatic genes that promote longevity. Understanding the mechanisms by which these bioactive compounds influence gene expression is crucial. For instance, polyphenols derived from extra virgin olive oil have been shown to inhibit the expression of the HER-2/neu protein, which is associated with aggressive breast carcinoma (46).

Moreover, this diet may increase cellular proliferation in endothelial progenitor cells (EPCs) and human coronary artery endothelial cells (HCAECs) and angiogenesis in EPCs, decrease intracellular reactive oxygen species (ROS) production and cellular apoptosis, and regulate the levels of miRNAs that are involved in the inhibition of genes and proteins related to biological processes associated with endothelial dysfunction and endothelial vascular homeostasis (47).

##### 3.2.2.2 DASH and epigenetics

The Dietary Approaches to Stop Hypertension (DASH) diet has been associated with beneficial epigenetic changes that may enhance cardiovascular health and metabolic function. A systematic review and meta-analysis indicated that adherence to the DASH diet significantly improves lipid profiles, reducing serum triglycerides and low-density lipoprotein cholesterol levels, critical for preventing cardiovascular diseases (48). The intake of potassium-rich vegetables and fruits, whole grains, poultry, fish, and nuts characterizes the DASH diet. It reduces sodium and saturated fat intake (49). Furthermore, research has shown that the DASH diet can lower high-sensitivity cardiac troponin I, a biomarker of subclinical cardiac injury, suggesting that dietary patterns may influence gene expression related to inflammation and heart function over time (50). These findings underscore the potential of the DASH diet in managing hypertension and promoting favorable epigenetic modifications that contribute to overall cardiovascular health.

In addition to its cardiovascular benefits, the DASH diet may also play a role in metabolic regulation through epigenetic mechanisms. Studies have highlighted that participants following the DASH diet experience improved insulin sensitivity and reduced metabolic risk factors linked to changes in DNA methylation patterns associated with obesity and diabetes (51, 52). For instance, a trial found that individuals on the DASH diet exhibited significantly improved blood pressure and metabolic markers compared to those on a standard American diet, suggesting that the dietary composition can lead to favorable epigenetic outcomes (53, 54). A systematic review and meta-analysis involving 2,218 individuals indicated that adherence to the DASH diet resulted in notable improvements in lipid profiles, including reductions in serum triglycerides and low-density lipoprotein cholesterol levels, which are critical for heart health (55). These results indicate that the DASH diet may serve as an effective intervention for enhancing metabolic health through epigenetic regulation, potentially reducing the risk of chronic diseases related to metabolic dysfunction.

##### 3.2.2.3 Low calorie diet and epigenetics

A very low-calorie diet typically limits daily calorie intake to < 800 kcal per day, aiming to promote rapid weight loss and improve obesity-related comorbidities (56). This diet involves partial or complete replacement of meals such as shakes, soups, or bars; it is recommended for ~8–16 weeks and under medical supervision (57). A randomized controlled trial showed that the weight loss stress accompanying a long-term calorie restriction accelerates telomere length attrition. However, this can be mitigated or ablated as new homeostatic norms are established; authors suggest that calorie restriction modulates core longevity pathways related to mitochondrial stability, inflammation, and oxidative stress (58).

A low-fat diet may slightly increase the content of endothelial progenitor cells (EPCs) in patients with non-severe endothelial dysfunction (47). A research project associated CpG sites' basal methylation with the reduction of Body Mass Index (BMI) percentage after 4 months of this dietary intervention. Individuals from Spain who were overweight and obese were assigned to a low-fat diet for 4 months. As a result, 20 CpG sites were identified in blood and were associated with a reduction in the percentage of BMI, specifically the methylation of the HLA-DRB5 gene. Conversely, the blood methylation of the WWOX gene showed a negative correlation with the decrease in BMI percentage. The hypermethylation of this gene is associated with adverse changes in metabolic health (59).

**Figure 4:**
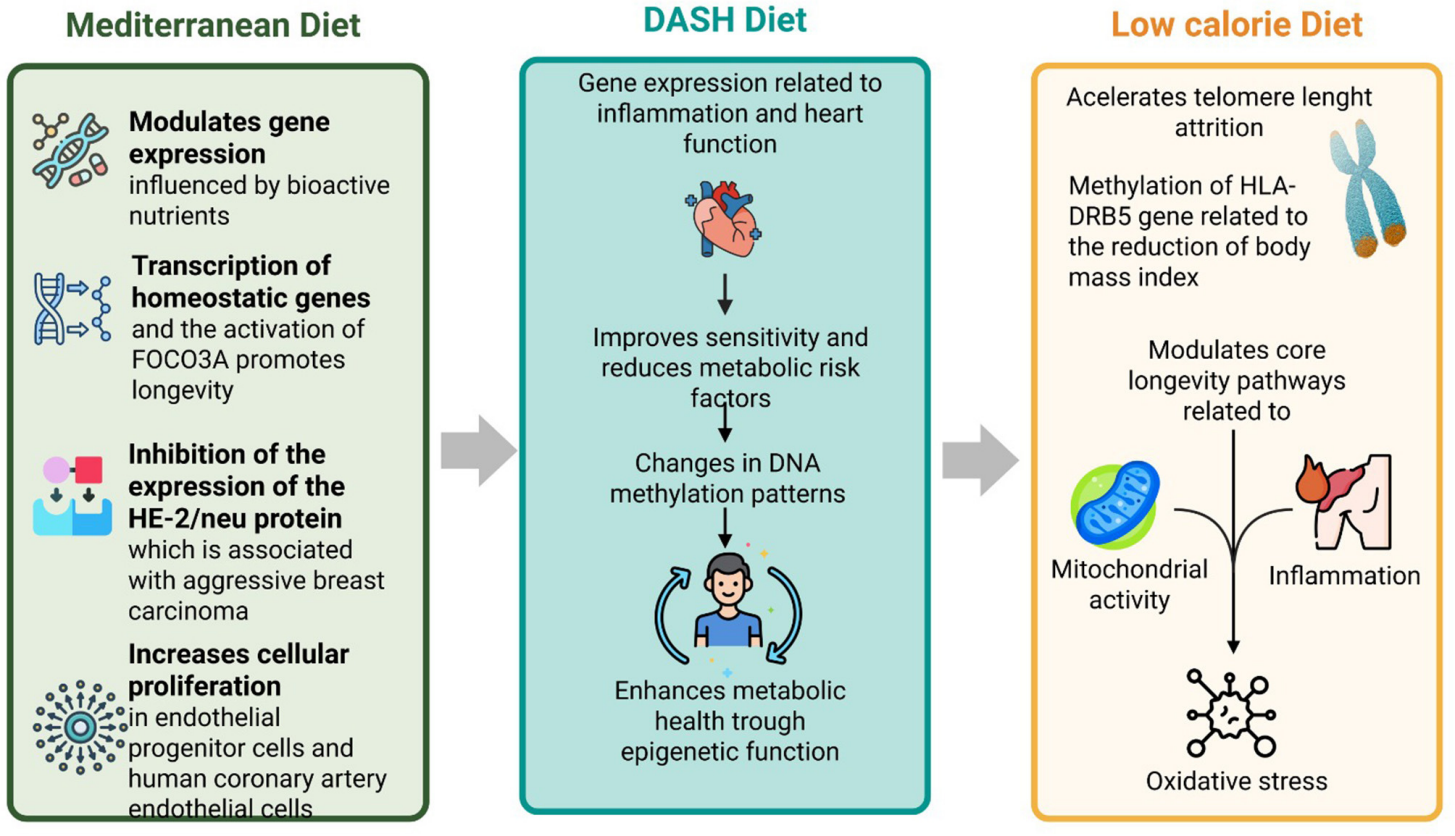
Influence of different types of diets and the epigenetic modulations.

Another type of dietary intervention is very low-calorie ketogenic; this diet is characterized by a very low carbohydrate content, 1–1.5 g/protein ideal body weight, 15–30 g fat/day, and ~500–800 caloric intake/day (60). In an epigenome-wide association study, 988 CpG sites showed differential methylation after this dietary intervention; those CpG sites encoded 786 genes involved in the function of adipose tissue and neurons, muscle development, and other metabolic processes. Additionally, global hypomethylation was observed in differentially methylated CpGs, which were previously identified as being correlated with downregulation of the DNA methyltransferase family. Changes in methylation levels of genes involved in the insulin pathways, collagen-related genes, and genes involved in adipocyte signaling were also observed (61) (Figure 4).

#### 3.3.3 Physical activity and epigenetics

Strength training, commonly referred to as weight training or resistance training, involves performing physical exercises to enhance muscular strength. While it is often associated with weightlifting, strength training encompasses a variety of techniques, including bodyweight exercises, isometric holds, and plyometric movements. Strength training offers numerous benefits, including improved metabolic homeostasis and positive adaptations across multiple tissues, which help mitigate age-related degenerative processes, particularly in middle-aged and older adults. It enhances mobility and contributes to disease prevention, supporting overall health and quality of life throughout the adult lifespan. Additionally, strength training is recognized as an effective intervention for reducing the risk of obesity and metabolic diseases. It promotes beneficial changes in adipose tissue, such as lowering adipocyte size, modulating pro-inflammatory adipokines, decreasing reactive oxygen species and inflammation, and increasing vasculature—processes that are often dysregulated in obesity. These effects make strength training a key strategy for managing insulin resistance and preventing metabolic dysfunction (62, 63).

High-Intensity Interval Training (HIIT) is a training approach that involves alternating between short bursts of vigorous exercise and brief recovery periods. These exercise segments are performed at “near maximal” intensity, which means they are vigorous but do not lead to total exhaustion. In the literature, the intensity levels are typically assessed using metrics such as maximal oxygen consumption (VO_2_ max, classified as high intensity ≥90), maximal heart rate (HR_max_, ≥77%), or maximal work capacity (W_max_, ≥80%). Among the benefits of HIIT, we can find the physiological changes that improve health; it enhances aerobic and anaerobic capacity, reduces blood pressure, boosts cardiovascular health, increases insulin sensitivity, optimizes cholesterol levels, and decreases abdominal fat and body weight while preserving muscle mass. Furthermore, HIT is versatile and time-efficient, making it accessible to individuals at various fitness levels and in different situations. It can incorporate a range of exercise modalities, including running, cycling, swimming, resistance training, and bodyweight exercises, and can also be effectively performed in group settings (64–68).

Hybrid training, also known as combined training (CT), concurrent training (CT), or strength and conditioning/endurance training, refers to an exercise regimen that trains both muscle strength and cardiorespiratory fitness within the same training cycle. This integrated approach typically combines resistance training with aerobic exercises to improve muscular and aerobic performance. The benefits of CT are well-documented, with evidence suggesting improvements in several physiological parameters, including explosive strength, VO_2_ max, aerobic capacity, endurance performance, maximal muscle strength, and muscle morphology. However, the effectiveness of CT compared to single-modality training remains a topic of debate. While some studies indicate that CT may provide superior benefits over isolated training methods, others have reported attenuated cardiovascular and musculoskeletal adaptations when both endurance and strength training are combined in the same session. Additionally, some research suggests that optimal improvements occur when strength and endurance exercises are performed in separate sessions rather than concurrently (69–72).

Physical activity has been shown to exert a complex and bidirectional influence on epigenetic mechanisms, leading to various beneficial effects on human health. One key aspect of this relationship involves epigenetic reprogramming, which can enhance global DNA methylation patterns and influence gene expression. For example, physical activity has been found to improve the age-dependent expression of the ASC gene, a regulator of interleukin production, thereby reducing the expression of pro-inflammatory cytokines. Additionally, exercise can induce histone modifications that regulate chromatin structure, thus influencing transcriptional activity and mRNA expression and affecting protein synthesis. Physical activity also impacts telomere dynamics, with some studies suggesting that exercise may either reduce telomerase activity or increase telomere length, both of which have implications for cellular aging and longevity. These epigenetic changes contribute to various physiological benefits, including improved insulin sensitivity and glucose metabolism, enhanced cardiovascular health, increased muscle strength and endurance, reduced inflammation and oxidative stress, and improved cognitive function and neuroprotection. Thus, physical activity improves health outcomes and induces lasting molecular changes that support long-term wellbeing (73–79).

##### 3.3.3.1 High-intensity interval training (HIIT) and epigenetics

High-intensity interval training (HIIT) is a form of exercise that involves short bursts of high-intensity exercise interspersed with recovery periods (80). It has gained popularity in recent years due to its effectiveness in improving cardiovascular fitness and metabolic health in a relatively short period. HIIT has been shown to improve glucose uptake and oxidation, increase insulin sensitivity, and reduce fat mass (81). The mechanisms by which HIIT affects metabolism are not fully understood. However, they are thought to involve a combination of factors, including increased activity of energy-sensitive signaling pathways, altered gene expression, and mitochondrial adaptations (80).

HIIT activates signaling pathways that promote mitochondrial biogenesis, resulting in the creation of new mitochondria (82). Mitochondria are the powerhouses of cells, playing a crucial role in cellular energy metabolism. By increasing the number of mitochondria in skeletal muscle, HIIT can improve the muscles' ability to produce ATP, the primary energy source for muscle contraction. HIIT also activates signaling pathways that enhance mitochondrial function (79). For example, HIIT has been shown to increase the activity of AMPK, a key enzyme that regulates energy metabolism. Activation of AMPK leads to several changes that improve mitochondrial function, including increased fatty acid uptake and oxidation (81). HIIT also induces gene expression in mitochondrial metabolism. For example, HIIT has been shown to increase the expression of PGC-1α, a transcriptional coactivator that plays a key role in mitochondrial biogenesis (83). Increased expression of PGC-1α leads to increased expression of genes involved in mitochondrial metabolism, resulting in improved mitochondrial function.

Therefore, High-intensity interval training (HIIT) triggers complex metabolic adaptations through multiple molecular pathways: activates key energetic sensors, such as AMPK and PGC-1α, which promote mitochondrial biogenesis and optimize oxidative metabolism (84); induces GLUT4 transporter expression, improving insulin sensitivity (85); increases mitochondrial density and function by activating NRF1/2 transcription factors (86); stimulates MAPK and CaMK signaling pathways that modulate gene expression related to energy metabolism (87); and generates epigenetic modifications that alter DNA, resulting in increased aerobic capacity and cellular metabolic efficiency (88).

The study demonstrated that high-intensity interval training (HIIT) induces significant changes in gene expression. RNA sequencing revealed that, out of 5,944 transcripts, 35 were differentially regulated after seven HIIT sessions. Eight of these transcripts were related to mitochondria. Those that experienced the greatest increase were mitochondrial creatine kinase 2 (CKMT2), mitochondrial pyruvate transporter (MPC1), cytochrome c oxidase subunit 7A2 (COX7A2), and mitochondrial ribosomal protein L41 (MRPL41). In addition, a gene set enrichment analysis (GSEA) identified 17 positively regulated cellular components, of which nine are related to mitochondrial remodeling. These components include the mitochondrial respiratory chain, the inner membrane, and protein complexes. The most positively regulated pathway was the mitochondrial respiratory chain, including genes such as COX7A2 and CYCS. Increases, although less pronounced, were also observed in transcripts of complexes I, II, III, and IV. These results point to a strong induction of transcriptional regulation, particularly in complex I genes, consistent with the improvement in respiratory function observed after HIIT (88). Similarly, these findings demonstrate that HIT is a potent stimulus to activate mitochondrial biogenesis through a mechanism involving nuclear accumulation of PGC-1α. This process leads to a rapid improvement in the oxidative capacity of skeletal muscle, which may have important implications for metabolic health (80).

This controlled trial, conducted by Schmitz et al. (89), explored the connection between HIIT, microvascular structure (especially the glycocalyx), and circulating miRNAs, proposing new tools to assess the cardiovascular benefits of exercise. Similarly, the findings indicate that high-intensity interval training (HIIT) may enhance insulin sensitivity by influencing PGC-1α and AdipoR1, revealing that metabolic improvements can be achieved even with only a slight decrease in body weight among overweight or obese individuals participating in HIIT (90).

##### 3.3.3.2 Hybrid training and epigenetics

Hybrid training, which integrates strength and endurance modalities, has garnered significant interest in the scientific community due to its multifaceted benefits on physical fitness and potential epigenetic modifications (7). This training approach not only enhances athletic performance but also appears to exert influence on the epigenome. This complex regulatory system modulates gene expression through chemical modifications without altering the underlying DNA sequence (91, 92).

According to Charles T. Putman and coworkers, their findings suggest that hybrid training, which combines strength and endurance stimuli, may generate a unique epigenetic profile in skeletal muscle. This profile could reflect a specific modulation of DNA methylation in genes related to muscle hypertrophy and mitochondrial biogenesis, changes in histone modifications that favor the expression of genes associated with slow twitch fibers and metabolic adaptation, and a particular regulation of the expression of miomiRs that control muscle cell proliferation, differentiation, and regeneration (93). In the same way, this article presents the results of the randomized clinical trial EFICAN, which investigated the effects of a 12-week supervised resistance training program combined with physical activity at home on fitness and quality of life in female breast cancer survivors. The study, with 60 participants, compared a resistance training group with a control group that performed only physical activity at home. A significant increase in total, upper, and lower muscle strength was found in the resistance training group, with a large effect size. However, no significant improvements were observed in other fitness components or subjective measures of quality of life, fatigue, or depression. The study emphasizes the importance of implementing resistance training principles correctly in exercise oncology clinical trials (94).

##### 3.3.3.3 Overall impact of physical activity on epigenetics

**Figure 5:**
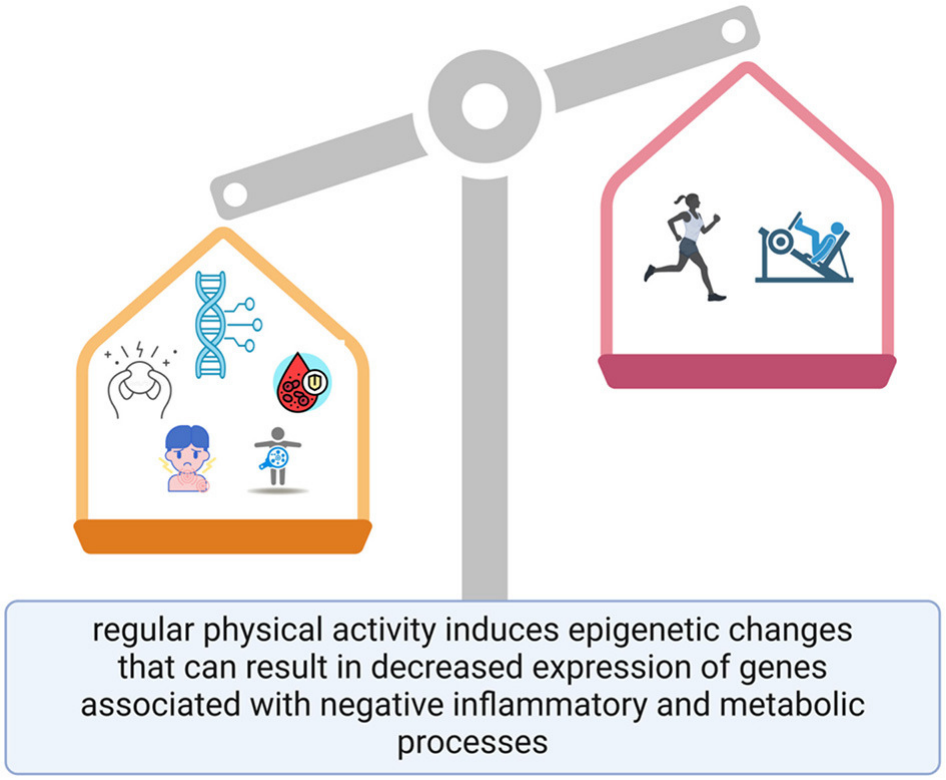
Relevance of physical activity and epigenetics.

Physical activity exerts a profound influence on human health, and its effects extend to the molecular level, affecting gene expression through epigenetic modifications (141). Exercise, whether endurance, strength, or a combination of both, acts as a potent stimulus that reshapes the epigenetic landscape, particularly in skeletal muscle. The sources provided explore the complex interactions between physical activity and epigenetic alterations, highlighting the role of DNA methylation, histone modifications, and microRNAs in shaping muscle adaptations. It is important to note that the impact of physical activity on epigenetic alterations is complex and depends on a variety of factors. The intensity and duration of exercise, the type of training (endurance, strength, or combined), and individual variability, influence the magnitude and types of epigenetic modifications (Figure 5).

Moreover, the impact of physical activity on epigenetics extends beyond individual health to influence intergenerational health outcomes. Studies indicate that parental physical activity can influence the epigenetic landscape of offspring through mechanisms such as alterations in the sperm epigenome (95, 96). This transgenerational transmission of epigenetic information suggests that lifestyle choices made by parents can have lasting effects on the health and wellbeing of their children (7, 96). Understanding the intricate relationship between physical activity and epigenetic modifications is essential for developing effective public health strategies aimed at improving population health across generations. As research continues to unravel these complex interactions, it becomes increasingly clear that promoting regular physical activity is vital not only for individual health but also for fostering a healthier future generation.

Strength training, like other forms of exercise, creates physiological stress that triggers adaptive responses. These responses are mediated by epigenetic mechanisms, such as DNA methylation in muscle cells, leading to activation or suppression of genes associated with muscle growth, repair, and metabolism. For instance, genes involved in muscle hypertrophy (like IGF-1 and myostatin) may experience changes in methylation status after resistance training (97). Histone modifications: strength training can induce histone acetylation, making genes more accessible for transcription. This can upregulate genes related to mitochondrial biogenesis, protein synthesis, and cellular repair. MicroRNA (miRNA) regulation: resistance exercise regulates muscle adaptation by affecting protein synthesis pathways (e.g., the mTOR pathway). Specific miRNAs such as miR-1, miR-133a, and miR-206 are associated with muscle differentiation and hypertrophy.

#### 3.3.4 Epigenetic mechanisms underlying lifestyle interventions: molecular targets and health implications

Recent evidence indicates that lifestyle interventions modulate key epigenetic processes that directly impact gene expression and molecular homeostasis. Several studies have not only detailed the general mechanisms of epigenetic modification but also identified specific genes affected, reinforcing the clinical potential of these strategies.

Regarding mindfulness, intensive meditative practices have been shown to induce relevant epigenetic changes. Studies have reported that meditation can influence the methylation of the NR3C1 gene (glucocorticoid receptor), involved in stress response, and the FKBP5 gene, which regulates cortisol sensitivity (98). Additionally, changes in genes such as IL6 (interleukin-6) and TNF-α (tumor necrosis factor-alpha) suggest a direct anti-inflammatory effect at the epigenetic level. These findings support the potential of mindfulness to modulate key signaling pathways associated with chronic stress and inflammation.

In the field of nutrition, multiple studies have demonstrated the impact of bioactive dietary compounds on critical regulatory genes. High intake of polyphenols has been associated with changes in the methylation of genes, such as DNMT1 (DNA methyltransferase 1) and SIRT1 (sirtuin 1), both of which are related to aging and metabolic regulation (99). Furthermore, the green Mediterranean diet, rich in polyphenols from sources such as green tea and Mankai, has been shown to promote the methylation of FOLR1 (folate receptor) and MTHFR (methylenetetrahydrofolate reductase), which are essential for DNA methylation and one-carbon metabolism (100).

Regarding physical exercise, intense activity such as high-intensity interval training (HIIT) induces stable epigenetic adaptations in skeletal muscle. These include changes in genes such as PGC-1α (peroxisome proliferator-activated receptor gamma coactivator 1-alpha) and PPARγ (peroxisome proliferator-activated receptor gamma), critical for mitochondrial biogenesis and lipid metabolism (101). Similarly, hypomethylation of the ASC gene (apoptosis-associated speck-like protein containing a CARD) has been observed, suggesting an improvement in inflammatory regulation (102). Additionally, the use of epigenetic biomarkers is emerging as a strategic tool for evaluating metabolic responses to lifestyle interventions. Specific epigenetic signatures involving genes such as IGF2 (insulin-like growth factor 2) and LEP (leptin) have been proposed as predictors of response to specific diets and physical activity programs (103). This paves the way for personalized interventions based on individual epigenetic profiles.

Altogether, current data support that lifestyle interventions directly influence the expression of key genes through specific epigenetic mechanisms. These modifications in genes regulating inflammation, metabolism, stress, and aging offer new opportunities to integrate preventive and therapeutic strategies based on molecular modulation.

## 4 Discussion

The assessment of long-term epigenetic effects remains a significant limitation in current lifestyle intervention research, with most studies focusing on short-term changes that may not accurately reflect sustained biological impacts. Modern lifestyles have a significant influence on epigenetic processes, which can, in turn, lead to various health issues. Understanding this relationship is crucial for advancing clinical studies aimed at mitigating lifestyle-related diseases. This is characterized by poor nutrition, sedentary behavior, and chronic stress, which have been shown to affect epigenetic mechanisms. For instance, the intake of energy-dense foods and exposure to environmental toxins can lead to epigenetic changes that disrupt normal metabolic functions, contributing to conditions such as obesity and cardiovascular diseases (104, 105). Therefore, research on epigenetic clocks has highlighted the need for better understanding of how these biomarkers respond to lifestyle interventions over time, particularly considering factors such as race, sex differences, lifestyle, and environmental influences.

Recent systematic reviews have consistently identified the temporal limitation as a major weakness in current epigenetic research. This temporal limitation is particularly problematic given that epigenetic modifications can be dynamic and subject to reversal upon cessation of interventions. Research by Chen et al. demonstrated that exercise-induced DNA methylation changes in muscle tissue showed significant attenuation at 6 months post-intervention, with ~60% of the initially observed modifications returning to baseline levels. This finding underscores the crucial need for sustained lifestyle interventions and extended observation periods to accurately assess the durability of epigenetic adaptations (106).

The challenge of maintaining consistent lifestyle interventions over extended periods introduces substantial methodological complexities that current research inadequately addresses. A longitudinal study utilized repeated measures of DNA methylation at FKBP5 and NR3C1 promoter regions, demonstrating that sustained practice was necessary to maintain hypomethylation patterns associated with improved stress resilience. However, the high attrition rate and varying levels of practice intensity created significant analytical challenges, limiting the ability to draw definitive conclusions about long-term epigenetic stability (107).

Current research methodology lacks standardized protocols for assessing the temporal dynamics of epigenetic modifications, particularly in distinguishing between acute, adaptive, and sustained changes. A systematic review on exercise interventions found that “Given the heterogeneity and complexity of the existing literature, it is currently not possible to propose a specific recommendation about the type, intensity, or duration of exercise that could be beneficial for different subsets of the population”, highlighting the methodological inconsistencies that plague the field (108). This temporal inconsistency complicates the interpretation of results and prevents meaningful comparisons across studies. The analysis revealed that acute epigenetic responses (measured within 72 h) often showed different patterns compared to chronic adaptations (measured after 3+ months), suggesting that early changes may not predict long-term epigenetic stability. The lack of standardized measurement protocols particularly affects studies of dietary interventions, where epigenetic changes may occur gradually over months rather than weeks.

A pilot randomized clinical trial demonstrated that natural aging processes result in systematic changes to DNA methylation patterns that can confound the interpretation of lifestyle intervention effects, particularly in studies with extended follow-up periods (109). This finding has profound implications for long-term lifestyle studies, as it suggests that current analytical approaches may not adequately separate intervention-specific effects from natural aging processes, particularly in older adult populations (110). Therefore, studies proposed that certain epigenetic modifications may require “priming” periods of months to years before becoming detectable, particularly those involving chromatin remodeling and establishment of new transcriptional programs (110). This concept is supported by research in developmental biology showing that environmental exposures can have transgenerational epigenetic effects that skip generations, suggesting that lifestyle interventions may have benefits that extend far beyond the intervention period (111). However, current study designs are not equipped to capture these delayed or transgenerational effects, potentially underestimating the full impact of lifestyle modifications on epigenetic health (112).

The inadequate consideration of seasonal and circadian variations in epigenetic measurements represents a significant source of unexplained variance in long-term studies that may obscure true intervention effects. Research demonstrated that DNA methylation patterns exhibit significant circadian rhythmicity, with up to 15% of CpG sites showing time-of-day dependent methylation levels (113). Additionally, seasonal variations in gene expression and epigenetic modifications, likely related to changes in daylight exposure, physical activity patterns, and dietary habits, can introduce substantial noise into longitudinal measurements (7, 44).

The limited integration of multiomics approaches in long-term lifestyle studies prevents a comprehensive understanding of how epigenetic changes relate to downstream molecular and physiological effects over time (114). While short-term studies increasingly employ integrated analyses of DNA methylation, gene expression, and metabolomic profiles, long-term studies rarely maintain this level of molecular phenotyping due to cost and complexity constraints. A review highlighted that only 8% of lifestyle intervention studies longer than 1 year included multiomics profiling, limiting our understanding of how epigenetic modifications translate into functional biological changes over time (115). This gap is particularly problematic for understanding the mechanistic basis of lifestyle-induced health improvements, as epigenetic changes may not immediately translate into transcriptional or metabolic alterations, requiring extended observation periods to capture the full cascade of molecular effects.

A study involving 52 women in Korea demonstrated that adherence to a Traditional Korean Diet (K-diet), which is rich in one-carbon nutrients from plant foods, resulted in increased global DNA methylation levels (116). Participants who followed the K-diet showed higher intakes of folate and other essential nutrients, leading to significant changes in plasma biomarkers, such as 5-methyltetrahydrofolate and L-homocysteine. The K-diet group exhibited a notable increase in global DNA methylation (from 6.70% to 9.45%, *p* < 0.0001), suggesting that this dietary pattern may have beneficial epigenetic implications for health and disease prevention. Another study involving 342 midlife women in the USA found that higher diet quality, as assessed by various dietary indices like the Alternate Mediterranean Diet (aMED) and the Alternate Healthy Eating Index (AHEI), was associated with decelerated epigenetic aging (117). Healthier diets correlated with lower levels of GrimAge, an epigenetic clock that estimates biological age based on DNA methylation patterns. This suggests that improved dietary quality can positively influence epigenetic markers related to aging and longevity.

A randomized controlled trial comparing a polyphenol-rich Green Mediterranean Diet to traditional Mediterranean and healthy dietary guidelines revealed that the Green-MED diet enhanced epigenetic regulatory potential, with thousands of differentially methylated regions identified (118). This indicates that specific dietary components can modulate gene expression through epigenetic mechanisms, further supporting the role of diet in health promotion. In the same way, a pilot study from the NU-AGE project investigated the effects of a 1-year Mediterranean diet on epigenetic aging markers. This study involved participants from various countries and assessed how adherence to the Mediterranean diet influenced global DNA methylation patterns (119). Results indicated that the diet led to significant epigenetic rejuvenation, with variations observed based on country and sex. The findings suggest that long-term adherence to a Mediterranean dietary pattern may positively affect biological aging processes through epigenetic modifications, highlighting its potential as a dietary intervention for promoting healthy aging. Another study examined how adherence to a Mediterranean diet during pregnancy affects the gut microbiota composition and epigenetic programming of offspring (120). Conducted with 33 pregnant women, the research utilized food frequency questionnaires to assess dietary patterns and collected biological samples from umbilical cord blood and placental tissue. The study found that mothers with higher adherence to the Mediterranean diet had offspring with distinct microbiota profiles and differentially methylated regions in genes associated with health. This suggests that maternal dietary choices can influence not only the immediate health of the mother but also have lasting epigenetic effects on their children, emphasizing the importance of dietary counseling for expectant mothers.

The Dietary Approaches to Stop Hypertension (DASH) diet has demonstrated significant effects on cardiovascular health and epigenetic regulation through various clinical studies. A randomized controlled trial assessed the DASH diet's impact on glycemic response and inflammation in obese patients with non-alcoholic fatty liver disease (NAFLD), revealing that participants on the DASH diet experienced significant reductions in inflammatory markers and improved metabolic profiles (121). Another study focused on pregnant women with pre-existing diabetes mellitus found that those following the DASH diet had lower gestational weight gain and potentially better perinatal outcomes compared to those on a standard diet, further supporting the diet's beneficial effects on health outcomes (122). Collectively, these studies highlight how the DASH diet not only aids in managing hypertension but may also induce favorable epigenetic changes that promote cardiovascular health.

Low-calorie diets have also been linked to beneficial epigenetic changes, particularly in relation to aging and metabolic health. A randomized controlled trial involving participants with abdominal obesity demonstrated that an 18-month hypocaloric diet led to significant alterations in DNA methylation patterns associated with aging biomarkers. Successful weight loss was correlated with a deceleration of epigenetic aging, indicating that caloric restriction can positively influence biological age at the epigenetic level (123). Furthermore, this article describes a randomized, controlled clinical trial comparing the effectiveness of a very low-calorie diet (VLCD) vs. a moderate energy deficit diet in obese women with polycystic ovary syndrome (PCOS) (57). The study found that VLCD resulted in greater weight loss and more pronounced improvements in body composition, hyperandrogenemia, and metabolic parameters than the moderate deficit diet.

Mindfulness is a practice that cultivates awareness of the present moment without judgment. Its importance lies in its multiple benefits at the psychological, physiological, and epigenetic levels. Several studies have demonstrated its effectiveness in reducing stress, anxiety, and depression, as well as in improving general wellbeing and quality of life. For instance, meditation programs during pregnancy have shown significant benefits for both the mother and fetal development, including reduced prenatal stress, anxiety, and depression, improved pain management during labor, and decreased obstetric complications (124). Mindfulness-based interventions during gestation help regulate emotional responses, improve maternal-fetal connection, reduce blood pressure and cortisol levels, and promote better sleep. Scientific studies have documented that regular meditation practice during pregnancy can positively influence fetal neurological development and reduce the risk of preterm birth. Additionally, the results of the study showed that Mindfulness-Based Stress Reduction (MBSR) significantly reduced prenatal stress and anxiety, but not depression (125, 126). Furthermore, the authors of the CALM Pregnancy pilot study concluded that Mindfulness-Based Cognitive Therapy (MBCT) has the potential to provide an effective, non-pharmacological treatment for pregnant women with anxiety; as well as Mindfulness-Based Childbirth and Parenting (MBCP) (127) program results were promising, with participants experiencing a statistically significant increase in mindfulness and positive affect, as well as a decrease in pregnancy-related anxiety, depression, and negative affect from pre-test to post-test (128).

Continuing in the same line, mindfulness practice has emerged as a promising intervention in the prevention and management of various diseases, supported by growing scientific evidence. Clinical studies have shown that mindfulness-based interventions can positively influence multiple biological mechanisms, including stress regulation, immune function, inflammatory processes, and gene expression. This research has documented significant benefits in the prevention and management of chronic conditions such as cardiovascular disease, diabetes, mood disorders, and autoimmune diseases, as well as in the reduction of risk factors such as hypertension, chronic stress, and obesity. The integration of mindfulness into preventive health strategies represents a promising non-pharmacological approach that can complement conventional medical treatments and improve patients' quality of life.

This pilot study evaluates the feasibility and acceptability of an online mindfulness intervention to reduce tics in six adults with Tourette Syndrome or Persistent Tic Disorder, seeking an accessible alternative to traditional treatment (129). A similar study evaluates mind-body therapy for chronic pain in patients with Ehlers-Danlos Syndrome through weekly group sessions that include meditation (130), in contrast, this study evaluates the benefits of The Mindfulness-Based Stress Reduction (MBSR) program in women newly diagnosed with breast cancer, measuring psychological and immunological outcomes, compared to a control group receiving health education (131). These three studies are related in the psychological domain, this one evaluates using the online mindfulness program “Aware” in 60 adolescent–parent couples with 22q11DS syndrome, comparing an immediate intervention group against a waiting list group (132), as well, this pilot study evaluates a modified mindfulness-based cognitive therapy to treat depressive symptoms and suicidal ideation in older adults, comparing with treatment as usual for 8 weeks and finally(133), this study examines the effects of mindfulness-based group therapy in patients with schizophrenia, evaluating changes in oxytocin levels, empathy and its relationship with genetic factors (134).

High-Intensity Interval Training (HIIT) has been shown to induce notable epigenetic modifications. For instance, a study conducted by Hsu et al. (3) revealed that HIIT leads to hypermethylation of the ACADVL gene, which is associated with reduced cardiac fibrosis in heart failure patients. This suggests that HIIT not only improves physical fitness but also has protective effects against cardiovascular diseases through epigenetic reprogramming. Similarly, research by Pilotto et al. (143) demonstrated that human skeletal muscle retains an “epigenetic memory” of HIIT, characterized by differential methylation patterns that persist even after a period of detraining. This memory is linked to increased gene expression related to calcium signaling and lactate transport, indicating that the benefits of HIIT may extend beyond immediate physiological adaptations. The concept of “transcriptomic age” has emerged as a novel metric for assessing biological aging. Lohman (144) found that participants engaging in HIIT exhibited a reduction in transcriptomic age by ~3.59 years compared to a control group that experienced an increase in transcriptomic age. This finding underscores the potential of HIIT as an effective intervention for mitigating age-related decline in health markers. Finally, this article mentions that HIIT's influence extends to gene expression related to inflammation and metabolic health. A study by Soltani et al. (145) highlighted that combined HIIT protocols significantly decreased inflammation markers in overweight and obese young women, suggesting that such training regimens can improve insulin sensitivity independent of changes in body composition. Additionally, Pashaei et al. (146) reported improvements in metabolic risk factors among middle-aged women undergoing HIIT, further supporting the role of exercise in enhancing metabolic health through epigenetic mechanisms. These discrepancies highlight the need for further investigation into the timing, sequencing, and frequency of combined training to determine the most effective protocols for enhancing both muscular and aerobic fitness.

One of the main limitations of studies on nutrition and genetics, especially in the context of dietary intervention, is the difficulty in controlling all the variables that can influence the results. Factors such as individual genetics, lifestyle, environment, and dietary adherence can affect the response to a nutritional intervention, making it difficult to determine the specific impact of diet. In addition, most studies rely on self-reported data on dietary intake, which may be unreliable. Finally, the interaction between multiple genes and diet is complex and requires larger and more sophisticated studies to fully understand their influence on health. Despite the evidence on the benefits of physical exercise, there are limitations in the studies investigating its impact at the molecular level. The lack of standardization in exercise protocols, individual variability in response to training, and the difficulty in isolating the effect of exercise from other lifestyle factors make it difficult to interpret the results. In addition, many studies are based on self-reported data on physical activity, which can be inaccurate. More research with robust designs and objective measures of physical activity is needed to better understand its influence on health and epigenetics. While mindfulness research has shown promising results, there are still limitations. The lack of replication of studies in independent cohorts, the use of self-reported stress data, and the difficulty in controlling all environmental variables during interventions are some of the limitations. In addition, the duration of studies is often short, making it difficult to assess the long-term effects of mindfulness. More research with longitudinal designs, objective measures, and mediation analyses is needed to better understand the mechanisms through which mindfulness impacts health.

The findings from recent studies on dietary patterns and lifestyle interventions provide valuable insights for practical applications in disease prevention and health promotion. For instance, in the NU-AGE study, adherence to the Mediterranean diet was assessed using a NU-AGE index scoring system based on 7-day food records. This suggests that integrating technology into lifestyle interventions can facilitate behavioral modifications and empower individuals to make healthier choices in real time. Furthermore, systematic reviews on diet modifications during cancer treatment highlight the importance of whole foods and exercise in improving treatment-related outcomes and quality of life for patients. These findings advocate for healthcare providers to promote healthy dietary patterns and physical activity as integral components of treatment plans, potentially mitigating adverse effects associated with cancer therapies. Additionally, understanding the epigenetic mechanisms influenced by lifestyle factors can inform personalized interventions aimed at optimizing health outcomes. By focusing on dietary quality, physical activity, and stress management, future research can develop targeted strategies that leverage lifestyle changes to enhance overall wellbeing and reduce the risk of chronic diseases.

## 5 Future research directions

Future research into how lifestyle factors impact epigenetics is poised to uncover significant insights into the mechanisms by which diet, exercise, and stress management influence gene expression and health outcomes. For instance, studies have shown that dietary patterns such as the Mediterranean diet can reduce neuroinflammation and improve cognitive function, suggesting a potential epigenetic modulation of genes involved in these processes. Additionally, the role of physical activity in enhancing metabolic health and reducing the risk of chronic diseases is increasingly recognized, with evidence indicating that exercise may induce epigenetic changes that promote cardiovascular health and improve insulin sensitivity. Furthermore, exploring the effects of psychological stress on epigenetic regulation could provide valuable information on how lifestyle interventions, including mindfulness and stress management techniques, can mitigate adverse health effects associated with chronic stress. As research continues to evolve, integrating multidisciplinary approaches will be essential for understanding the complex interplay between lifestyle factors and epigenetic mechanisms, ultimately guiding personalized health strategies aimed at disease prevention and health promotion.

Part of our research focused on lifestyles, including outdoor activities, and their impact on epigenetics. Although we did not find results specifically on this association, we discovered findings on the relationship between greenness and epigenetics. However, we chose to exclude these findings as they fall outside the scope of our study. Nevertheless, investigating the association between greenness and epigenetics, particularly in relation to diseases, would make for a promising research topic. Similarly, we examined modern lifestyles, such as veganism and the ketogenic diet, in the context of nutrition. However, we had to exclude these topics due to the limited number of available articles. The relationship between these dietary interventions and epigenetics warrants further investigation and would be a significant focus for future studies.

Ultimately, it is essential to emphasize the importance of future research utilizing advanced technologies to enhance our understanding of the molecular alterations triggered by lifestyle modifications. These changes occur across a wide range of tissues and organs, and by examining the underlying pathways involved, we can enhance the precision and effectiveness of interventions. Expanding these same research interventions beyond genomics to incorporate multiomics, such as metabolomics, epigenomics, proteomics, and single-cell RNA sequencing, will provide a more comprehensive view of how lifestyle modifications impact various biological systems. By systematically investigating these multiomic layers, we can identify which specific changes are most impactful in preventing or managing chronic diseases, thereby prioritizing the lifestyle interventions that produce the most significant health benefits or even targeting a more personalized and effective strategy.

The systematic exclusion of emerging dietary approaches such as veganism, ketogenic protocols, and intermittent fasting from epigenetic research represents a significant limitation that may be overlooking important therapeutic targets and mechanistic insights. Recent systematic reviews on nutrition and epigenetics have noted significant gaps in research coverage, particularly for plant-based diets and alternative dietary patterns that are gaining clinical relevance. The limited scope of current dietary research may reflect publication bias or insufficient research infrastructure rather than a lack of biological plausibility. Research by Dwaraka and coauthors, a twin study, shows that an 8-week vegan diet reduces epigenetic age acceleration compared to an omnivorous diet, highlighting anti-aging effects and specific DNA methylation changes linked to improved metabolic health and reduced inflammation (135). However, this research remains preliminary and lacks the depth of research seen in more established dietary patterns. The limited research on ketogenic diet-induced epigenetic modifications represents a particularly notable gap given the diet's growing therapeutic applications and distinct metabolic effects. This study shows how the ketogenic diet improves metabolic health by altering adipose tissue function, enhancing lipid metabolism, modulating UCP1 and FGF21 pathways, and influencing epigenetic mechanisms linked to weight loss and inflammation (136).

Another limited investigation of sleep optimization interventions represents another significant gap in lifestyle epigenetic research, despite extensive evidence linking sleep quality to gene expression and cellular health (137, 138). This study evaluated that the sleep-disordered breathing is linked to faster epigenetic aging, with stronger effects in women for arousal index and in men for hypoxia-related acceleration, and the exclusion of social and community-based lifestyle interventions from epigenetic research overlooks the potential importance of social connections and collective behavior change in modulating gene expression (139, 140). New research focus has emerged highlighting the interactions between social factors and health. The term *social determinant of health* often refers to any non-medical factor that directly affects health, including values, attitudes, knowledge, and behaviors (140).

## 6 Conclusion

Physical activity is a powerful tool for modulating the epigenome and improving health. Exercise induces changes in DNA methylation, histone modifications, and miRNA expression, which orchestrate muscle adaptations and confer health benefits. Understanding the complex interactions between physical activity and the epigenome is crucial for designing personalized exercise strategies that optimize health and performance. Hybrid training represents a promising approach to enhance physical fitness while potentially influencing the epigenome. As research continues to explore these connections, understanding how combined strength and endurance exercises affect gene regulation will be crucial for developing optimized training protocols tailored to individual health objectives. Further studies are needed to elucidate the specific epigenetic mechanisms in response to hybrid training regimens.

Mindfulness practices not only improve mental and physical wellbeing but also demonstrate potential for influencing epigenetic mechanisms such as DNA methylation, histone modifications, and non-coding RNA activity. Studies reveal that mindfulness interventions can modulate stress-related gene expression, enhance immune function, and mitigate biological aging. These findings highlight mindfulness as a valuable approach for promoting health by addressing both psychological and molecular pathways. On the other hand, nutritional interventions, including adherence to diets such as the Mediterranean diet, DASH diet, and a low-calorie diet, play a significant role in shaping epigenetic modifications that influence health outcomes. Nutrients such as polyphenols, omega-3 fatty acids, and carotenoids can modulate gene expression through DNA methylation and histone modifications, reducing the risks of chronic diseases such as cancer and cardiovascular disorders. These findings underscore the importance of diet in promoting health and longevity through epigenetic regulation.

As seen in our research, lifestyle factors, such as physical inactivity and poor dietary habits characterized by the consumption of ultraprocessed foods, and chronic stress are strongly associated with the development and progression of chronic diseases. These are the leading causes of death worldwide, affecting individuals across all age groups, genders, and ethnicities. With the results exposed here, we wanted to highlight the role of lifestyle modifications at the genomic and proteomic level and their clinical significance, not only in the prevention of these diseases but also in improving outcomes for those already diagnosed. Lately, in clinical practice, Lifestyle Medicine (LM) focuses on addressing the root causes of chronic conditions by prescribing lifestyle changes as a form of “medicine” alongside or in place of conventional drug treatments. By integrating evidence-based lifestyle interventions—such as improved nutrition, increased physical activity, stress management, and sleep optimization—into clinical practice, LM aims to enhance patient health, prevent disease progression, and improve the management of chronic illnesses. This approach emphasizes the importance of personalized care, where the modification of lifestyle factors becomes a central therapeutic strategy for both disease prevention and treatment.

## Supplementary material

Supplementary Table 1 An analysis of 14 research studies revealed various epigenetic mechanisms associated with mindfulness interventions. The primary epigenetic modifications observed included DNA methylation changes, particularly at specific CpG sites, telomere dynamics, and histone modifications. Key findings showed altered DNA methylation profiles in experienced meditators, with one study identifying changes at 61 CpG sites after intensive meditation. Several studies have documented gene alterations related to the stress response, including those affecting COMT, FST, FKBP51, and MAOA. Additionally, research demonstrated changes in histone deacetylase genes (HDAC 2, 3, and 9) and pro-inflammatory gene expression among meditation practitioners. The studies encompassed diverse populations, from medical students to individuals with specific health conditions, with intervention periods ranging from 1 day to several months.

Supplementary Table 2 An analysis of 25 research studies revealed diverse epigenetic mechanisms associated with different exercise interventions, primarily involving changes in DNA methylation, miRNA expression, and transcriptomic alterations across various populations. High-intensity interval training (HIIT) demonstrated significant transcriptomic age reduction (3.59 years) and DNA methylation changes in genes related to oxidative stress and cardiac function. In contrast, combined training (aerobic + resistance) showed modulation of genes, including BRCA1 and SLFN12, with notable changes in PGC-1α methylation and miRNA expression related to mitochondrial biogenesis. Resistance/strength training revealed modifications in genes involved in metabolism (GPAM, SREBF1, and SREBF2) and inflammation (IL-6, TNF-α), with studies documenting exercise-induced changes in enhancer activities and methylation patterns in metabolic pathways. The research encompassed diverse populations, ranging from sedentary adults to individuals with specific health conditions, with intervention periods varying from single sessions to 14-week programs. This research consistently demonstrated that exercise interventions can induce significant epigenetic modifications, which may contribute to improved health outcomes through various molecular mechanisms.

Supplementary Table 3 The studies demonstrated a diverse range of dietary interventions, including the Traditional Korean Diet, which is high in plant foods and low in calories. Mediterranean diet variants (including a green version rich in polyphenols and reduced in red meat), DASH diet, Very-Low-Calorie Diet (VLCD), high-protein/low-carbohydrate diets, and vegetarian diets. The intervention durations varied significantly, ranging from 3 months (as in the Mediterranean vs. vegetarian diet study) to 18 months (in the Green-MED study). Study populations were diverse, including women only in some studies, mixed populations in others, and specific groups such as obese individuals or those with Polycystic Ovary Syndrome (PCOS). Despite the variety in intervention types and durations, the studies consistently showed improvements in various health markers, particularly in epigenetic measurements such as DNA methylation patterns, changes in gene expression, and biological aging markers. For instance, the Korean diet increased global DNA methylation, the Green-MED diet revealed 1,573 differentially methylated regions, and the DASH diet was associated with lower levels of epigenetic aging markers, such as DunedinPoAm, GrimAA, and PhenoAA.
